# Open-Air Treatment in Great Britain: A Survey and a Criticism

**Published:** 1903-04-25

**Authors:** T. N. Kelynack

**Affiliations:** Assistant Physician to the Mount Vernon Hospital for Consumption and Diseases of the Chest, Hampstead and Northwood, London


### Open-Air Treatment in Great Britain: A Survey and a Criticism.

By T. N. KELYNACK, M.D., M.R.C.P., Assistant Physician to the Mount Vernon Hospital for Consumption and Diseases of the Chest, Hampstead and Northwood, London.

The importance of the urgent need for a rational application of hygienic methods in the treatment of disease is daily becoming more generally recognised. Influences long accepted as desirable for the maintenance of a high degree of what we are pleased to term health are now known to be of even greater importance in assisting the victim of disease to a satisfactory restoration.

The benefits of the so called "open-air" treatment of disease, especially in the case of consumption, have been so unmistakable that the attention both of the public and the profession has been arrested, and the subject is receiving much serious consideration in its hygienic, medical, economic, social, and other bearings.

The present occasion seems opportune for a survey and a criticism of the situation. I venture, therefore, on a brief restrospect, and make bold to present some features of a forecast.

Pulmonary tuberculosis or consumption of the lungs is a malady of great antiquity, the clinical characters of which have been recognised since the days of Hippocrates. It is not surprising, therefore, to find the great medical reformation of modern times, if we may call it so, centering around this ancient scourge of humanity.

In the investigation of disease, as in the study of man, an application of the evolutionary method has thrown considerable light on many perplexities, and afforded directing energy which has enabled us to break the bonds of tradition and loose the fetters of authority, and make free excursion along paths which experience and experiment now clearly show are leading to fields where science is capable of providing means for almost limitless expansion.

The modern methods of pathological investigation have unlocked the doors to new kingdoms, and we are but beginning to learn the application of our newly acquired knowledge to the needs of suffering mankind.

In all ages man has diligently groped in the darkness for an explanation of the maladies which circled him by despair, and finally crushed him by death, and although gleams of the coming dawn have occasionally brought hope and invigoration to the pioneers, it is but recently that we have made near approach to the mysteries of the ætiology of disease. Through the discovery of the actual causes of the ills which beset us we are being granted an insight into the most effectual means for their prevention and arrest. It is well to realise that the modern awakening in regard to the application of hygienic measures in the treatment of consumption occurred in this country.

Mr. George Bodington, "Surgeon," as he called himself, of Sutton Coldfield, near Birmingham, in 1840 published his now classic work entitled "An Essay on the Treatment and Cure of Pulmonary Consumption on Principles Natural, Rational and Successful; with Suggestions for an improved plan of treatment of the disease amongst the lower classes of society; and a relation of several successive cases restored from the last stage of consumption to a good state of health."

In this monograph he attacked the then prevalent, irrational and non-hygienic management of consumptives in a manner so vigorous that it is well to recall the writer's own words:—

"I believe having mentioned the shutting-up plan in close rooms, the use of antimony and digitalis, if I add the use of demulcents, of blisters, leeches, plasters, etc., I shall have described the helpless and meagre system of medical treatment of consumption in general use at the present day, the utter uselessness of which is so well known and so obvious that the members of the medical profession in the towns are in the habit of dismissing their patients to some distant seaport or watering-place, where, falling under precisely the same mode of treatment, they there die. The gravestones in the churchyards of many of these places of resort of the consumptive patients bear testimony to the truth of this remark."

And later this true pioneer clearly indicates his firm grasp of the truth and righteousness of open-air methods:—

"To live and breathe freely the open air, without being deterred by the wind or weather, is one important and essential remedy in arresting its progress, one about which there appears to have generally prevailed a groundless alarm lest the consumptive patient should take cold."

It is unnecessary to linger over the record of the slow advance towards a full acceptance of the importance of a rational application of hygienic treatment, but the work of such pioneers as Bodington, MacCormac, Bennet, Brehmer, and Dettweiler should always be accorded honoured recognition. And in the foundation of the pathological principles on which our modern treatment is in great measure based, the names of Villemin and Koch must ever be remembered with respect.

### Principles of "Open-air" Treatment.

Consumption is now often declared to be one of the most curable of the chronic infectious diseases. The pessimism of the past has given place to a well-founded and thoroughly practical optimism. Attempts at alleviation are now supplemented by efforts carefully designed and scientifically maintained for the absolute arrest of the disease, and the restoration of the sufferer to the ranks of the workers.

Unfortunately, however, the support accorded to open-air methods by a large section of the profession and the public generally is, in great measure at least, founded on a faith without works, a belief without knowledge, a trust without trial. It is most necessary that every practitioner should by personal observation make himself fully acquainted with the principles, and as far as possible the details, of sanatorium treatment.

It should be clearly understood that in the "open-air" method, as conducted in modern sanatoria, we have no "specific" treatment for consumption, and that an abundant supply of pure air is only one of many hygienic necessities for the restoration of the phthisical subject. In the light of modern research old ideas as to "predisposition" are being recast. We prefer now to express our meaning in terms indicative of degrees of tissue resistance. There is much, however, still to be made clear before we can fully understand the nature of such powers, estimate their strength, and satisfactorily provide for their establishment and maintenance. But this much is definite that in the adoption of so-called "natural" methods of treatment we can most efficiently provide those conditions which shall permit of the highest development of the body's powers of resistance to deranging influences.

Instead of "open-air" methods, many prefer to speak of "hygienic" treatment, and no doubt such a term is more accurate. But whatever be the mere expression we may decide to adopt, the great objects are :—

1. To remove the patients from the sphere of action of the causal agents of the disease.
2. To secure conditions which shall allow of the development of the highest powers of resistance.

### The Factors in Hygienic Treatment.

To Brehmer belongs the honour of being the first to adequately systematise the essential factors in the hygienic treatment of consumption. He insisted on the importance of—

1. Open-air life under such natural conditions as provide immunity from tuberculosis.
2. Freedom from debilitating circumstances or influences which may excite exacerbation.
3. Methodical hill climbing.
4. Abundant dietary.
5. Various hydrotherapeutic measures.
6. Constant medical supervision.

These requirements have undergone considerable modification in detail, but the essential features remain supported both by experience and experiment.

At the present time chief insistence is made on the employment of—

1. Continuous exposure to open air.
2. Abundant sunlight.
3. Carefully regulated rest.
4. Graduated exercise.
5. Abundant feeding.
6. Strict compliance with all hygienic requirements.
7. Constant medical supervision.

In connection with hygienic treatment other measures and certain drugs are used in some sanatoria. In many institutions the employment of medicaments is practically discarded. There can be no doubt that in some cases at least carefully selected agencies are of considerable service in assisting purely natural methods, but there is reason to believe that in many instances absolute harm is done, and restoration hindered by an ignorant and meddlesome use of drugs, fashionable exercises, various electrical appliances, and other much lauded agencies.

The exploitation of phthisis-therapy unfortunately is a particularly favourite and profitable pursuit for unscrupulous quacks.

### The Advantages of Sanatorium Treatment.

Experience has clearly shown that the application of hygienic measures in the treatment of consumption can be best accomplished in a specially-constructed, suitably-equipped, and carefully-conducted institution, and hence in almost all civilised countries sanatoria are now quickly springing into being. The benefit of treatment in such special establishments arises from many causes which need not now be discussed in detail.

But while every experienced physician is probably fully convinced of the overwhelming advantages of the disciplined and carefully-directed life of a sanatorium, I consider it but short-sighted policy to decry or even minimise the service of treatment by natural measures carried out at home under the supervision of a well-informed and thoroughly-conscientious practitioner.

Not a few of my out-patients while waiting their turn for admission to a sanatorium have made very considerable progress in the direction of arrest. A fairly considerable experience in the post-mortem room of cases of "healed phthisis," has also fully convinced me that a very considerable number of subjects successfully fight their way back to complete restoration, even under circumstances lacking in almost all the essentials of our smodern methods. But the admission of such facts, instead of discouraging the adoption of a rigorously conducted hygienic treatment seems to me to supply some of the strongest arguments in its favour.

### The Results of Sanatorium Treatment.

Satisfactory data regarding the results of sanatorium treatment are particularly difficult to obtain. There is a lack of anything like a convenient and uniform method of classification. At all times it must be difficult to eliminate the influence of the personal equation, but there is urgent need of at least an attempt to arrive at and honestly record results as they really are. At the present time the results are most discordant. Some private sanatoria claim, as Dr. Arthur Latham has recently shown, that over 80 per cent. of their early cases attain apparent arrest of the disease, and even some place 100 as indicating the total percentage of cases in which there was real improvement; while, on the other hand, in some free institutions the satisfactory results are estimated at figures as low as 4 per cent. for apparent arrest and 19·5 for real improvement.

Careful discrimination should be made between pathological cure and economic arrest. To secure absolute obsolescence generally requires a much longer time than any patient is willing to spend, or indeed can financially afford, in a sanatorium, but the hygienic manner of life initiated in such an institution when continued in a rationally-conducted home or residence other than a specially-constructed sanatorium can undoubtedly in very many instances secure such economic cure as will allow the patient to return to the ranks of the workers and under certain conditions obtain a competency.

To speak without qualification of "cure" is misleading and mischievous. I venture to think that a practical cure should not be claimed unless after two years discharge from a sanatorium a patient is free from evidences of active disease and following, or able to follow, an occupation which shall afford him at least a living wage.

Dr. S. Moritz in a recent endeavour to ascertain the results of treatment finds that of 83 patients discharged as improved from the Manchester Hospital for Consumption, where hygienic methods are as far as possible employed, 36 per cent. succumbed or had become worse.

It is particularly desirable that at least every public institution undertaking the treatment of the consumptive should furnish an annual record as to its results.

The recently issued medical report of the Mount Vernon Hospital, prepared by Dr. S. R. Williams and approved by the Medical Board furnishes an attempt in this direction. After giving statistics of the cases treated, detailing the nature of the methods followed, indicating the character of dietary and the like, particulars are presented regarding the benefit or otherwise attending the treatment.

The incidence of pulmonary tuberculosis on the various trades and occupations is shown by an analysis of 465 cases, from which it appears that a little over 80 per cent. of the cases were engaged in work necessitating an indoor life, while less than 20 per cent. worked out of doors. Although under the pressure of circumstances many advanced cases were admitted, the death-rate was only 1·5 per cent., and of the eight fatal cases five were admitted in a moribund condition and died within ten days, while two of them died on the day of admission; the whole five came up from various country districts and ought never to have been allowed to take the journey.

Of 460 cases of phthisis treated by open-air methods 66·5 per cent. left the hospital "much improved" or "improved," the former registering an average gain per week of over a pound. As regards the extent and stage of the lesions 46 09 per cent. of the patients had two lobes affected, while in only 14·7 per cent. was the disease limited to one lobe. In 68 early cases in which the evidences of involvement was limited to one upper lobe 92·7 per cent. underwent "much improvement" and "improvement." An analysis of the influence of family history in 460 cases went to show the importance of a tuberculous family history in increasing the gravity of the prognosis.

It is clear that if reliable information is to be secured as to the immediate and remote results of sanatorium treatment a fairly uniform and thorough method of compiling returns should be adopted. At the present time we are very far from this. In every sanatorium a careful record of cases should be kept. In some institutions the resident medical staff is not sufficiently large to meet such very necessary demands.

### Requirements of a Good Sanatorium.

There is still much difference of opinion as to the best form of sanatorium, and the most convenient manner of providing for its conduct. The matter is attracting much attention and is now receiving serious study in this country.

Dr. A. G. Welsford and Mr. A. W. S. Cross, in a recent communication, express the opinion that an ideal sanatorium should meet the following requirements:—

1. Free play of sunlight upon each room and block of buildings provided for the patients' accommodation.
2. The freest possible circulation of air around each building, and the avoidance of all enclosed spaces in which air would be liable to become stagnant.
3. Ample space between the various buildings.
4. Facilities for enabling the patients to spend the greater part of each day in the open air.
5. Sufficient compactness of plan to allow of all departments being placed within reasonable distance of the administration centre, which should be separated from the patients' quarters.
6. Isolation of the engine and boiler-house, the laundry, mortuary, and other subsidiary buildings.
7. Departmental separation of male and female patients, whose quarters and exercise grounds should be placed on either side of a central administration block.

The recent competition in connection with the scheme for the King's Sanatorium has given rise to much activity in connection with the preparation and discussion of designs for such institutions.

There seems at the present time to be too great a tendency, mainly, it is true, on the grounds of economy and administrative convenience, to aggregate many cases in a large, single, and often two or more storied, building. The worst type of sanatoria is undoubtedly that constructed on the "barrack" system, while the ideal is that of the "separate cottage." The establishing of a cottage colony is necessarily expensive, both as regards construction as well as administration, but it cannot be doubted that its advantages are such that, in the near future, at least some approximation to such a plan will be attempted. The herding together of large numbers of tuberculous cases in one large institution is strongly to be condemned. In many sanatoria the patients either in wards or Liegehallen are permitted such constant, close approximation that it is practically impossible to insist that the respiratory products of one case shall not prejudicially influence another. I am of opinion that in the future we shall come to recognise the importance of extending the "cottage system." Of this we may be sure when the State recognises the necessity of providing for those members of the community who have been permanently handicapped by a tuberculous invasion, garden cities or consumptive colonies will have to be established in which such huge sanatoria as are now springing up in different parts of the country will have no place, but where the unit will be the cottage.

### Cost of Establishing and Maintaining a Sanatorium.

Only brief references are here necessary to the most important financial considerations of the subject.

The question of estimate of cost can only be satisfactorily considered when site, situation, size, and all the particulars in regard to a proposed sanatorium are available. Mr. A. G. R. Foulerton in association with Mr. R. Langton Cole have recently published a scheme for a model pavilion sanatorium approximating to the cottage system and designed to accommodate 100 cases, and they have furnished an estimate of cost which is at least sufficiently suggestive to be worthy of record, and I therefore venture to present their interesting summary:—

| | |
|---|---|
| Buildings, including drainage and all machinery, plant and fittings, but not furniture ... ... ... ... | £90,000 |
| Furniture, including linen, etc. | 3,000 |
| Road making and laying out grounds ... ... ... ... | 2,000 |
| Farm buildings ... ... ... | 10 000 |
| Land (say 100 acres) ... | 10,000 |
| Balance left for endowment | 85,000 |
| Total ... ... | £200 000 |

The income can be raised as follows:—

| | | |
|---|---|---|
| 3 per cent. on ... ... ... | £85,000 = | £2 500 per annum. |
| The payment of 88 poorer patients might average 8s. per week, or £20 per annum : 88 × £20 ... | = | 1,760 per annum. |
| Twelve better class patients might pay 30s. per week or £78 per annum : 12 × £78 ... ... ... | = | 936 per annum. |
| Total ... | | £5,246 |

This would give an average income of £1 per patient per week which would probably be just sufficient for the ordinary expenses of the sanatorium.

There seems to be an idea among some that a sanatorium can be conducted very much on the lines of a convalescent home. In certain recent estimates for the nursing staff of a public sanatorium an altogether inadequate allowance has been made. Unless these institutions are conducted in accordance with strictly scientific procedure and under constant medical supervision, aided by an adequate nursing staff, disappointment and disaster will inevitably follow.

### Sanatoria : Demand and Supply.

Although pulmonary tuberculosis is undoubtedly declining in this country, it is still accountable for an enormous amount of suffering and a terrible waste of life.

It is estimated that at the present time in England and Wales alone there is an annual mortality of 40,000 persons from consumption ; and between the ages of 15 and 25 one fourth of the total deaths are said to be due to this disease.

Dr. J. Milson Rhodes has recently estimated that one-eleventh of the pauperism of this country results from phthisis alone, and that the working-class of to-day is losing from ten to eleven million pounds sterling per annum from the ravages of this disease.

At the present time the sanatoria accommodation available is insignificant and altogether inadequate to meet the enormous demand.

It is very difficult to arrive at anything like a reliable return as to the number of beds available for the necessitous consumptive.

It must be remembered that many of the so-called "hospitals for consumption" are also hospitals for "diseases of the chest," and admit other pulmonary affections besides those of a tuberculous origin, as well as cases of heart disease. Mr. W. J. Morton has been at much pains to provide me with statistics which I venture to think may fairly be taken as a near approximation to the actual condition of affairs.

From these returns it would appear that at the present time in the United Kingdom for the free accommodation of consumptives in sanatoria constructed and conducted on modern lines there are less than 150 beds available. The special hospitals of London providing free accommodation for such cases have something like 665 beds at their disposal ; while those in the provinces and Scotland have together only about 78 beds.

Of course, in this return I am not considering the general hospitals, many of which still continue to admit phthisical cases, although there is a growing reluctance to provide for them. Some few Poor-law infirmaries are making attempts to afford some measure of open-air treatment for a few of their cases. But for the vast majority of the poor and struggling workers who are smitten by pulmonary tuberculosis there are no means of supplying adequate hygienic treatment.

The special hospitals and sanatoria admitting paying cases at rates at all within the limits of members of the working class have about 400 beds. And it must be remembered that some of these cannot offer by any means ideal conditions for open-air treatment.

The private sanatoria intended for well-to-do cases, and those capable of paying two and a-half guineas and upwards weekly, can provide for upwards of 500 cases.

It is true the present altogether meagre and woefully inadequate accommodation will soon be augmented by sanatoria which are in course of construction, but it will be long before the supply of suitable institutions for the necessitous consumptive will in any way meet the enormous demand.

At the present time I am told that in connection with our Mount Vernon Hospital we have 260 cases waiting admission ; this means that some patients cannot be admitted for more than five months ; and of all cases who apply for admission only about 60 per cent. are considered in any way suitable for sanatorium treatment

It is becoming abundantly clear that we must not rely solely on voluntary effort and the fluctuating support of the charitable and benevolent in the establishment of sanatoria for the indigent class and those who are competent only so long as they remain in full work.

Among recent figures bearing on this point, reference may be made to those of Dr. S. Moritz, who contends that at the present rate of cost of sanatoria, say £500 per bed to provide for 50,000 patients, would embody an original capital outlay of £25,000,000, and with an allowance of £1 per week per bed, an annual expenditure of $2\frac{1}{2}$ millions. Such an outlay is altogether beyond the possibilities of philanthropy.

There seems also to be no likelihood of public opinion in this country favouring the establishment of institutions conducted under any such system as that of workmen's insurance as practised in Germany. Of this we may be sure the question is of such vast proportion that if it is to be met effectually it will be necessary for the State to undertake the treatment of the necessitous consumptive and provide at the public expense suitable sanatoria for the cure of the curable and adequate retreats for the care of the incurable.

It is probable that the provision of adequate institutions for the consumptives of London might be best dealt with by the Metropolitan Asylums Board. Certainly on scientific as well as administrative grounds such a body seems most fitted to deal with an infective disorder like consumption.

In the provinces the county councils or district sanitary authorities would probably be the most suitable for the conduct of such work. In any case, and whether voluntary or compulsory notification be generally adopted or not, it is most desirable that the medical officer of health of a district should be in intimate touch with the authorities directing all sanatoria within his area.

### Selection of Cases.

From the above melancholy presentation of present "ways and means," it is manifest that to obtain the maximum of benefit in the minimum of time, cases for sanatorium treatment should be selected not only with regard to individual needs, but after very careful consideration of the actual necessities of the situation.

Having recently dealt with this matter in a communication to the Hunterian Society of London, I may perhaps be allowed here to make reference to certain conclusions there presented.

Concisely summarised the most favourable cases for sanatorium treatment are :—

Full-grown, well-built adults, with normal development of chest, good breathers, without antecedent disease, of healthy stock, and trained in such occupations as do not necessarily expose to non-hygienic condition. A calm, hopeful, orderly, and obedient patient makes the happiest inmate of a sanatorium.

The continuance of menstruation is generally considered a good sign.

Many cases in which the onset is acute do well, and oftentimes the lesion becomes limited and restoration is effected more rapidly than in many cases where the invasion is insidious and the progress slow. Cases in which pleurisy and hæmoptysis are among the initial manifestations often do remarkably well. Not a few cases in which there is slight laryngeal involvement prove very encouraging. I am inclined to think that some sanatoria make a mistake in not paying greater attention to the local treatment of lesions of the larynx.

The chances of recovery, generally speaking, may be said to be in direct proportion to the period at which detection of the morbid condition has been made.

The actual extent of the pulmonary lesion is a factor of considerable moment, but too much importance must not be placed on the so-called "stage" of the disease.

Among the unfavourable cases are :—

Subjects below puberty and of advanced age. (Children are generally best treated in special sanatoria and a sea-side situation seems peculiarly advantageous.) Low class town dwellers, ill developed, with misshapen chests and deficient respiratory powers, of reckless habits or undisciplined manner of life, with bad family history and giving evidence of previous lung enfeeblement and general deficiency in vigour, form a large section of the applicants for sanatorium relief, and are a peculiarly disappointing class. Subjects, and I think particularly females, who break down at or about puberty often do poorly even under the best hygienic conditions. Excitable, irritable, or morose neurotics are wont to be troublesome under the necessary rigorous discipline of a sanatorium.

Patients in whom tubercle has been implanted on an old chronic bronchitic and emphysematous base generally prove unsatisfactory ; indeed, cases in which signs of bronchial involvement persist I am always inclined to regard as most unfavourable for such open-air methods as are generally available in this country.

Where there is widespread cavitation the benefit gained is usually but briefly retained. Extensive disease of the lungs is distinctly unfavourable. Continuous high temperature, profuse sweating, uncontrollable cough, marked dyspnœa, tendency to cyanosis, intermittent attacks of pleurisy, persistent anorexia, dyspepsia, troublesome diarrhœa, insomnia, nervous irritability with despondency or melancholia are usually evidences which go far to make patients unsatisfactory subjects from the standpoint of the public sanatorium.

I am accustomed to consider a persistently rapid, irregular and low tension pulse a bad sign.

Phthisical patients the subjects of alcoholism almost invariably prove unsatisfactory ; and the subjects of diabetes, cardiac lesions, Bright's disease, or other serious organic affection, generally gain no permanent benefit by residence at a sanatorium.

A considerable number of persons who seek relief have been the subjects of syphilis, but unless there are active syphilitic lesions present there is no reason why such should be excluded.

Where there is profound anæmia, considerable toxæmia, general debility, extensive laryngeal mischief, fistula in ano, or evidence of a more generalised tuberculosis, the outlook is usually most serious.

Generally speaking examination of the sputum, although of course of great diagnostic value, affords but little assistance in the actual selection of cases.

Considering the difficulties of the present situation in affording suitable accommodation for desirable cases I am inclined to think that it is expedient, and even—considering the patients themselves—judicious, to exclude at once from our free public sanatoria all pauper aliens and such indigent subjects as will probably be compelled speedily to seek asylum in the workhouse infirmaries.

But a word of warning is very necessary. While we attempt to discriminate between those cases which are likely to react favourably to a properly applied sanatorium treatment, and those which will obtain no permanent benefit from hygienic methods it is very necessary that we should not be altogether led by mere generalisations. Oftentimes what appears to be the most favourable case fails to profit, while what seems the most unfavourable disappoints our fears. In fact every case should be studied individually and considered on its own merits.

It is customary for an applicant seeking admission to a sanatorium to present a report containing more or less definite clinical data. It would be well if the authorities of such institutions could realise that in the majority of instances these certificates, as generally filled in, are of but little value.

I think it would also be wise if medical men responsible for the conduct of public institutions dealing with consumptives, would boldly declare that in very many cases it is impossible to state with anything like certainty what the course of a case may be by merely one examination in an out-patient department, often after the patient has been fatigued by a long journey, wearied by much waiting, excited by the unaccustomed surroundings, and frequently depressed by the presence of strangers and other circumstances.

### Classification of Patients.

Pulmonary tuberculosis is a very variable disease. In certain forms and in particular subjects it is an eminently curable affection, while in some varieties, and when affecting subjects having feeble powers of resistance, it is one of the most hopeless of diseases.

There is particular need for a wise discrimination in this matter. It is unnecessary to insist on the special value of the so-called pathological "stages." Oftentimes a patient's chance of recovery is much better with a contracting cavity than with a small but extending patch of early infiltration.

Moreover, it must not be forgotten that modern research has clearly demonstrated the importance of recognising the influence of secondary infection in hastening the destructive processes in the lungs, lowering the vital powers and often determining a fatal issue.

The point I wish to insist on is that more adequate means than those now usually adopted should be found for a rational grouping of patients in sanatoria. In some institutions advanced and even moribund cases lie in close association with those which are incipient and improving. This is to be deplored and should be avoided : it is neither scientific nor humane.

On pathological as well as clinical grounds a grouping of cases is most desirable.

By adopting a grading of cases it is possible to introduce a very valuable factor of much personal encouragement and emulation, which is of the greatest assistance in maintaining a vigorous *esprit* among patients.

Those having the responsibility of the care of the phthisical should be fully cognisant of the main features of the psychology of the consumptive.

Some still seem to think that a sanatorium is a place where dangerous patients suffering from an incurable malady may be comfortably and conveniently isolated. As far as possible the essentially curable cases should not be allowed to associate with the apparently incurable. Institutions for the alleviation of the sufferings of advanced and hopeless cases should be kept quite distinct from the sanatorium proper. At the present time it would be well if special wards in connection with already existing hospitals could be set aside for incurable cases. The proposal to use the unused wards of isolation hospitals for advanced cases is in many ways objectionable, and is certainly not likely to be generally approved by the profession or favoured by the public.

### Extension of Open-air Methods to Other Diseases.

The encouraging results obtained by the treatment of consumption by hygienic methods, particularly when carried out in properly-conducted sanatoria, has gone far to arouse attention to the necessity of securing for all cases inherently feeble, or where by the neglect of compliance with physiological requirements or the establishment of actual disease, the powers of resistance to morbid influences are subnormal, suitable hygienic conditions which will permit of the highest possible development of recuperative powers and the establishment and maintenance of a stable equilibrium which shall allow free play to the exercise of the body's protective mechanism.

Before the results of recent pathological investigations had secured to us a sound scientific basis for a systematised treatment along these lines, experience had to some extent indicated the path of progress, and the prophylactic virtues of travel, the advantages of outdoor pursuits in certain climates, and the benefits of an unfettered life at sea, on the veld, in the bush, were clearly recognised. The establishment of seaside institutions and other health stations for the scrofulous and convalescent, and holiday homes for the debilitated have all pointed the same lesson. Even the experience of asylums and like institutions situated in healthy districts and usually allowing considerable opportunity for open-air life has clearly proved the physical advantages of a resort to natural measures. And yet until recently most of our general hospitals have been slow to recognise, or at least to admit, the advantages which a situation permitting free exposure to pure air must have over that of the city and town. The present movement for the decentralisation of London hospitals, and the tendency to move such institutions into more open districts, or at all events provide accommodation for those cases which can be moved into the country, is in some measure the outcome of a more general recognition of the value of open-air methods.

The light thrown on the pathology of many diseases now known to be infective or arising from toxæmic conditions, or dependent on states of lowered tissue vitality, also points strongly in the same direction. Syphilitics in an early stage are best treated medically when enjoying an open-air existence, and Dr. E. H. Douty has recently strongly advocated sanatorium life for such. Other of the granulomata are also advantageously treated by a like method.

In the management of many affections in which a morbid condition of the blood is the most conspicuous feature, like chlorosis, pernicious anæmia, certain secondary anæmias, purpura, various toxæmic conditions, the hygienic life made possible by a sanatorium will probably be found to greatly increase our effectual control.

"Open air" treatment is undoubtedly of the greatest service in all septic conditions. It is one of the best of antipyretic agents. We are gradually coming to see that many cases of small-pox, typhus and typhoid are advantageously controlled by treatment in the open air, and it is likely that many of our present fever hospitals, before many years are passed, will be considered altogether obsolete institutions for the best management of such cases as they now accommodate.

### General Considerations.

Open-air treatment has come to stay. It is fashionable, but fashion is sometimes based on science. It is very necessary, however, that between hindering exaggeration on the one hand, and utter apathy and helpless scepticism on the other, a sound and rational study of the question should be made by all having the true welfare of the people at heart, and particularly by those desiring an effectual alleviation of the present sad lot of the consumptive poor.

There is danger that fads and fancies may darken counsel, and the intrusion of selfish interests may cause some deviation from essentials.

In dealing effectually with consumptives, I am convinced that we should aim for some such arrangement as we are endeavouring to provide in the case of the Mount Vernon Hospital :—

1. A central out-patient department for the observation and selection of new patients, the treatment of such cases as cannot obtain admission to a suitable institution, and the periodical examination of those who, having undergone sanatorium treatment, have returned home or gone to work.

2. A hospital conducted on sanatorium lines in close touch with city life, where cases of acute onset may be located, and where doubtful cases may be watched and thoroughly investigated.

3. A country sanatorium where carefully selected cases may, under strict medical control and the best possible hygienic conditions, be directed to a practical recovery.

At the present time there is also urgent need for the establishment of suitable homes, such for instance as the excellent Friedenheim Hospital at Swiss Cottage for advanced cases, and the founding of colonies for those permanently crippled.

At present, perhaps, too much attention is centred in the sanatorium. In only too many instances but little effort is made in attempting any really effective application of hygienic methods in the home treatment, which, as we have shown, at the present time, is the only place available for many. By a little serious effort much might be done to educate the patient and the patient's friends in the details of the modern hygienic treatment of consumption, and by constant supervision secure a salutary carrying out of the same. Much quiet educational work goes on in many out-patient departments of hospitals, but nurses, district visitors and others brought into intimate contact with the poor, if trained in the application of open-air methods might accomplish much. Unfortunately, in only too many instances, after a certain experience of and advantage from sanatorium life has been gained, through the neglect of an efficient after-care, many cases are allowed to pass into an avoidable relapse. Ignorance and apathy in this, as in so many other matters, bar progress. Some have advised the establishment of camps near our cities and towns for the use of consumptives capable of continuing their work. It is well, however, to remember that most tents are exceedingly difficult to ventilate efficiently, and this is especially so in wet weather, and hence, probably, ordinary tents make, on the whole, far worse habitations than even a city room with open windows.

If there is to be a comprehensive campaign against pulmonary tuberculosis, and an extension of Municipal and State control in the management of indigent consumptives it is clear that from the administrative point of view notification is very desirable if not actually essential.

Some recent writers have suggested the adoption of somewhat radical measures. They advise compulsory notification of phthisis, and isolation where the medical officer of health deems it necessary of advanced cases in a suitable sanatoria or special hospital ; or if this be not at once possible a strict public health supervision of cases left in their homes, and an insistance that any advanced case should occupy a separate room. They also urge the necessity for an adequate increase of the staffs of all medical officers of health to be specially devoted to the supervision and control of tuberculosis ; and the authorisation to medical officers of health to appropriate small-pox hospital accommodation for cases of phthisis wherever in their opinion such accommodation exceeds the demands at all likely to be made by the occurrence of small-pox.

It is wise to remember, however, that many of our measures for dealing with consumptives are still but little advanced beyond the experimental stage ; and there is also good reason to believe that many of the above suggestions would not yet be welcomed by the profession, and they are certainly very likely to stir up considerable opposition among a large and important section of the public. If effectual advance is to be maintained it is manifest that it can only be done by practically the unanimous support of all medical men and sanitarians and a loyal compliance on the part of the public.

The leavening influence of education in regard to this matter is rapidly extending, and any precipitate action which shall add to the burden of the consumptive, increase the anxiety and distress of his friends or alienate the sympathy of the public is to be deplored and avoided ; for any measure having such an effect would almost infallibly bring about a reaction in popular sentiment which would seriously hamper and hinder all efforts at scientific reform.

Opportunities for the extension of the campaign against consumption are practically limitless. Much is being accomplished by the educational efforts both of the platform and the press. The upspringing of sanatoria under public control, or at all events with the support and cordial assistance of the most influential members of the public, affords a striking and ever-present object lesson. Every inmate of a sanatorium should be so trained as to become a preacher of the gospel of hygiene to his own immediate circle and a consistent practiser of the s[illegible]e in his own home.

It is not only into the dwellings of the poor that light and air must be brought, but also into the dark and heated apartments of the wealthy. Indeed, in regard to an intelligent perception of the value of the simple hygienic life the most cultured often manifest the profoundest ignorance. The artist, even more than the artisan, shuns exposure to cold, and the student is often among the most sceptical as to the prophylactic and curative influences of fresh air.

The modern awakening to the need and wisdom of seeking scientifically to secure the prevention and cure of consumption is a striking evidence of that silent process of evolution which is slowly but surely working for the betterment of the human ; and although almost insuperable difficulties seem at present to circle the subject, yet very real advance is being made, and it may well be that in the years to come sanatoria will take their place in history with leper houses and such institutions as, having well served their purposes, are allowed honourable remembrance among the things that have been.

